# Informing mHealth and Web-Based Eating Disorder Interventions: Combining Lived Experience Perspectives With Design Thinking Approaches

**DOI:** 10.2196/38387

**Published:** 2022-10-31

**Authors:** Hannah K Jarman, Siân A McLean, Rachel Rodgers, Matthew Fuller-Tyszkiewicz, Susan Paxton, Beth O'Gorman, Emily Harris, Adrian Shatte, Katie Bishop, Tahlia Baumann, Danielle Mahoney, Melissa-Claire Daugelat, Zali Yager

**Affiliations:** 1 School of Psychology Deakin University Geelong Australia; 2 Centre for Social and Early Emotional Development School of Psychology Deakin University Melbourne Australia; 3 School of Psychology and Public Health La Trobe University Melbourne Australia; 4 Applied Psychology Program for Eating and Appearance Research Department of Applied Psychology Northeastern University Boston, MA United States; 5 Department of Psychiatric Emergency & Acute Care Lapeyronie Hospital Centre Hospitalier Regional Universitaire Montpellier Montpellier France; 6 Melbourne School of Psychological Sciences University of Melbourne Melbourne Australia; 7 Department of Planning, Performance & Analytics James Cook University Townsville Australia; 8 School of Psychology University of Queensland Brisbane Australia; 9 Department of Psychosomatic Medicine and Psychotherapy Medical University Hospital Tübingen Tübingen Germany; 10 Centre of Excellence for Eating Disorders University of Tübingen Tübingen Germany; 11 Institute for Health and Sport Victoria University Melbourne Australia

**Keywords:** eating disorders, app-based intervention, lived experience, design thinking, interviews, young women, co-design, mobile health, mHealth

## Abstract

**Background:**

App-based interventions designed to prevent and treat eating disorders have considerable potential to overcome known barriers to treatment seeking. Existing apps have shown efficacy in terms of symptom reduction; however, uptake and retention issues are common. To ensure that apps meet the needs and preferences of those for whom they were designed, it is critical to understand the lived experience of potential users and involve them in the process of design, development, and delivery. However, few app-based interventions are pretested on and co-designed with end users before randomized controlled trials.

**Objective:**

To address the issue, this study used a highly novel design thinking approach to provide the context and a lived experience perspective of the end user, thus allowing for a deeper level of understanding.

**Methods:**

In total, 7 young women (mean age 25.83, SD 5.34, range 21-33 years) who self-identified as having a history of body image issues or eating disorders were recruited. Participants were interviewed about their lived experience of body image and eating disorders and reported their needs and preferences for app-based eating disorder interventions. Traditional (thematic analysis) and novel (empathy mapping; visually depicting and empathizing with the user’s personal experience) analyses were performed, providing a lived experience perspective of eating disorders and identifying the needs and preferences of this population in relation to app-based interventions for eating disorders. Key challenges and opportunities for app-based eating disorder interventions were also identified.

**Results:**

Findings highlighted the importance of understanding and identifying problematic eating disorder symptoms for the user, helpful practices for recovery that identify personal values and goals, the role of social support in facilitating hope, and aspects of usability to promote continued engagement and recovery.

**Conclusions:**

Practical guidance and recommendations are described for those developing app-based eating disorder interventions. These findings have the potential to inform practices to enhance participant uptake and retention in the context of app-based interventions for this population.

## Introduction

### Background

Eating disorders are serious, complex, and potentially fatal conditions. Up to 18.6% of women and 6.5% of men experience a diagnosed eating disorder in their lifetime, a figure that has increased over the past 18 years [1], particularly throughout the COVID-19 pandemic [2]. Subclinical or prodromal and clinically diagnosed eating disorders are associated with high levels of medical and psychological comorbidities and poor quality of life and have one of the highest mortality rates among all mental disorders [3-5]. Alongside considerable health costs for individuals and their families, eating disorders also substantially impede productivity, with the cost of disease burden for eating disorders each year estimated at Aus $69.7 billion (US $43.6 billion) in 2012 [6] and approximately US $64.7 billion in 2021 [7].

Early response and treatment are recommended to ensure the best outcomes for individuals [8]; however, there are system-wide and individual-level issues that prevent detection, recognition, diagnosis, and access to treatment for eating disorders [9,10]. Alongside the stigma associated with eating disorders, symptomatic individuals may not realize the severity of their symptoms or may be afraid of change, hence obstructing help seeking [9,11]. Depending on geographic location, health systems vary widely in relation to pathways, costs, and access to diagnosis and treatment [11,12]. Combined, these factors mean that as few as 25% of individuals with eating disorder seek help [13], one of the lowest treatment-seeking rates across mental disorders [14].

Self-help psychoeducational and treatment resources, delivered via the web through digital health platforms (ie, accessed via an app or computer), represent an opportunity for cost-effective access to intervention in a convenient and private manner. These approaches have demonstrated promise in the field of psychiatry (eg, depression and anxiety) [15] and specifically within eating disorders [16,17]. Studies of web-based self-help interventions for eating disorders report moderate acceptability and accessibility [18], with some evidence suggesting that they may be at least as effective as in-person interventions [19,20]. Despite the potential and realized advantages of digital health interventions, limitations exist. Individuals may be ambivalent to change [9] or express concerns regarding data privacy and quality of content within eating disorder apps [16]. For these reasons, mental health and eating disorder apps typically experience low engagement and high attrition rates [15,19].

Although these concerns are present in digital health more broadly, this study will focus specifically on mobile health. Given that app development as a whole is resource-intensive, researchers are encouraged to use user engagement practices to ensure that these investments translate to outcomes [21], particularly given the high levels of attrition in many digital health interventions. Focusing and prioritizing efforts to determine user needs and meeting them in app development will ensure that this investment of time and money will result in increased uptake and use. Although studies have started to explore eating disorder users’ preferences [16], reviews have concluded that studies involving end users in the design of such apps is lacking [19] and the few examples that exist do not adhere to best practices (eg, not using succinct and clear communication to end users [22]).

Incorporating voices of lived experience through participatory research and co-design is recognized as being critical in the intervention design process [23]. Design thinking is a novel approach for creative problem-solving, which involves empathizing with future end users, defining the problem, ideating and testing early concepts, and iterating until the product meets accessibility and usability needs [23-25]. This study focused on empathizing with potential end users of eating disorder apps and identifying possible challenges and opportunities within this population.

Empathizing with potential future users is the first phase within all design thinking frameworks. This is typically done by interviewing individuals with lived experience of the condition or topic that is being addressed. The purpose is to better understand the nature of their experiences, implications of the problem, and their attitudes toward possible solutions. Following the interview, the researcher independently develops an empathy map for each user to visually depict the user’s personal experience, including what they see, hear, say, do, think, and feel. Then, these are used to elicit deeper values, needs, and motivational drivers that may inspire more innovative solutions [23,26]. Although similar in structure to more traditional qualitative analysis, empathy maps are advantageous in that they provide empathy regarding what the individual sees, feels, and so on, thus allowing the researcher to contextualize participant data relative to the individual’s lived experience, rather than coming from the perspective of the researcher. In addition, the empathy mapping approach can be used to identify both challenges faced by the population of interest on a particular issue (ie, pain points) and possible opportunities, including how to overcome these obstacles (ie, gain points). Therefore, empathy maps allow for better understanding of the context, problems, and needs of the population, while also providing opportunities for addressing the topic or issue.

Design thinking approaches, such as empathy interviews and empathy maps, may be especially helpful when considering individuals with significant body image and eating concerns, including eating disorders, who often have a fear of change or losing control and have ambivalent attitudes toward change [9]. Empathy maps may contribute to knowledge gain regarding users’ wants and needs and inform the marketing and dissemination of these apps to address barriers and increase uptake, retention, engagement, and efficacy. Design thinking approaches have been used to inform technology-enabled eating disorder services [27-29] and in the design of a body image and eating disorder prevention program for adolescent boys [30]. However, no study has yet conducted an in-depth examination of the lived experience of individuals with eating disorders to inform app-based intervention development.

### Objectives

Given the opportunities that design thinking may provide [31], this exploratory study aimed to use a novel empathy mapping process to provide insights into end users’ perspectives of app-based interventions for eating disorders, including identifying key challenges and opportunities of app-based interventions among this population.

## Methods

### Recruitment

The study forms part of a large project (Web-Based Interventions to Reduce Eating Disorders [WIRED]), which involves the development and evaluation of an eating disorder app. Participants were recruited via posts on the WIRED project’s social media accounts, highlighting the value of sharing lived experience, focus on involving end user experiences in app development, and nature and aims of the project. In some instances, these posts were also reshared by partner organizations (eg, the Butterfly Foundation). Young adults were asked to express their interest in participating in the study if they were aged 18 to 35 years, lived in Australia, and had experienced body image issues or eating disorders. Recruitment focused on young adults because (1) eating disorders are prevalent in this age group and (2) this group is likely to be the highest users of an app. Therefore, *lived experience* was self-identified and not reliant on past or current formal diagnoses. Individuals who have not received an eating disorder diagnosis but nonetheless experience significant symptoms are at high risk of not receiving appropriate treatment and are therefore a key group that the to-be-developed app aims to engage. In an adjacent study, young adults were invited to participate in a quantitative survey, in which they were asked to provide demographic details and respond to questions focused on their needs and preferences for an eating disorder app (results presented elsewhere; refer to study by Daugelat et al, unpublished data, May 2020). However, participants who were interviewed for this paper did not necessarily complete that questionnaire.

### Participants

Young Australian women (N*=*7) who identified as having lived experience of an eating disorder or significant body image concerns provided informed consent to participate in this study. Given the exploratory nature of this study, the sample was not intended to be representative; therefore, a small sample size was deemed acceptable [32]. Furthermore, this sample size is aligned with the number of participants typically engaged in empathy interviews for user experience studies [33]. Of the 7 women, 1 (14%) woman chose not to provide demographic details; therefore, these are reported for the remaining participants (6/7, 86%). This 86% (6/7) of the participants was aged between 21 and 33 (mean 25.83, SD 5.34) years. Of the 6 participants, 5 (83%) lived in Melbourne, Victoria, and 1 (17%) lived in regional New South Wales. All except 1 participant had received a formal eating disorder diagnosis from a health professional (5/6, 83%) and had engaged in treatment, either previously (5/6, 83%) or currently (2/6, 33%).

This study adopted a combined methodology, which incorporated a design thinking approach with traditional qualitative interview research. The semistructured interview schedule is provided in Multimedia Appendix 1. The interviewer (female, Masters of Health Psychology student, aged 22 years, and with recent experience of a subclinical eating disorder) initially asked participants to share their experience of disordered eating and significant body image concerns and how it affected them personally. Following this, questions focused on the web-based environment and the development and use of digital resources such as apps. The aim of the interviews was two-fold: to hear the story and body image or eating disorder journey of the participant and to ask participants what they would have needed or wanted from an app-based intervention around that time.

### Ethics Approval

The Victoria University Human Research Ethics Committee approved all the procedures for conducting this research project (HRE 20-010). Participants provided active consent before completing a web-based survey to collect their demographic and contact details.

### Procedure

Interviews were conducted through the web via Zoom and were transcribed using web-based transcription software. Participants were provided a Aus $50 (US $30) gift card to thank them for their time and for sharing their lived experience.

### Data Analysis Strategy

Empathy mapping is the first stage in the design thinking process [23]. Using the interview transcripts, an empathy map was created for each participant to empathize with the individual by viewing the world through their eyes and visually articulating what is known about them and their experience through four key questions: what did they see, what did they say and do, what did they hear, and what did they think and feel. This process provides context with which one can reflect on the deeper needs of individuals, as expressed by the individuals, rather than coming from the perspective of the researcher. Given that the timeline for app development on the project was short, empathy maps were initially drafted by the interviewer and then extended to provide more detail by the lead author (female, aged 29 years, and with no lived experience of an eating disorder). The last author (female, aged 39 years, and with lived experience of a subclinical eating disorder), who is experienced in both design thinking and eating disorder research, ensured congruence between these stages.

Next, to ensure a lived experience perspective, an empathic lens was used to interpret the interview data, and inductive thematic analysis was performed [34]. This process meant that the lead author used the empathy maps to inform the interpretation of the data, to ensure that the lived experience of the participants was front of mind during the analyses, rather than their own perspective. These analyses were focused on the data that were relevant to intervention and recovery, specifically app-based interventions. An iterative, reflexive process was used, meaning that ideas were formed and developed over time. The lead author identified the initial themes [34]. Then, these were shared with the authors and multiple members of the research team (who were in the early stages of content development for the app). Following discussion and further development, themes were finalized by the lead author.

Alongside each theme, a single pain and gain point was identified to encapsulate key considerations regarding the challenges faced (ie, pain points) and how apps may overcome these issues (ie, gain points). Although pain and gain points traditionally form part of the empathy mapping process, given that the aims of the paper were focused on app-based interventions, interpretation of the pain and gain points was focused specifically on app-based interventions.

## Results

### Empathy Maps

**Figure 1:**
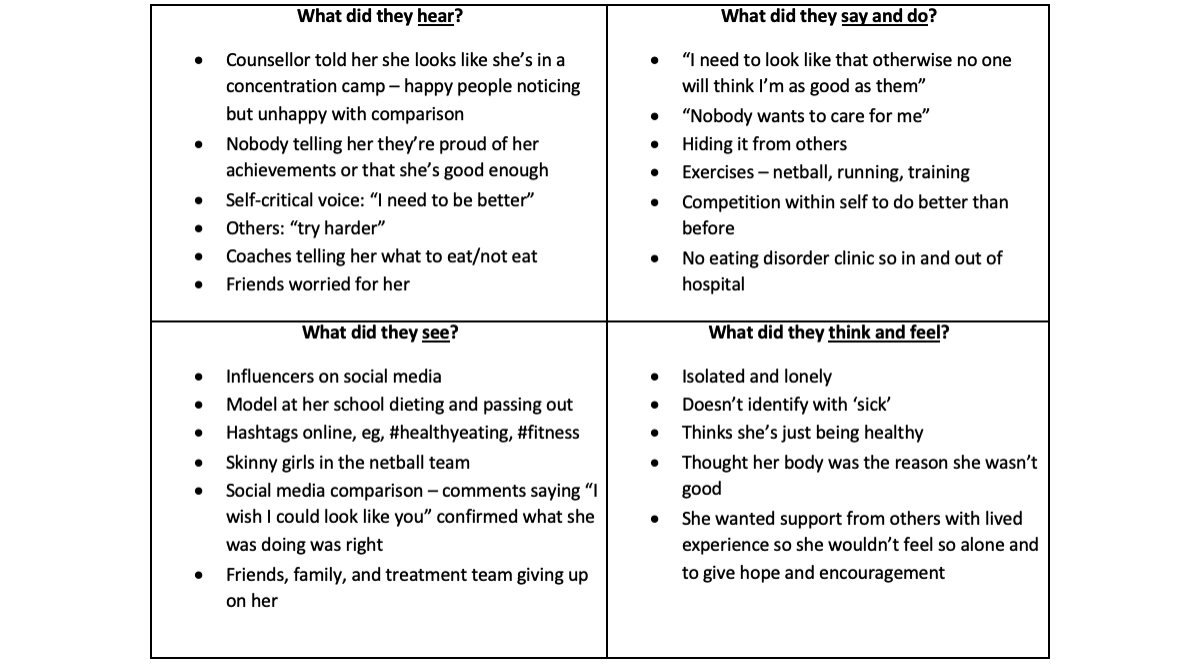
An example empathy map, which identifies the lived experience of 1 participant.

An example of an empathy map from a participant is shown in Figure 1. As described previously, the aim of this process was for the lead author to ensure that the lived experience perspective was considered during the thematic analysis. Furthermore, given that the purpose of the study was focused on app-based interventions rather than describing the lived experience (which is presented in detail elsewhere [35,36]), an overview of the common elements across the empathy maps is provided in Multimedia Appendix 2.

### Thematic Analysis and Pain and Gain Points

#### Overview

Following the empathy map process and attending to this lived experience perspective, 4 themes were identified relating to insights into the needs and preferences for app-based eating disorder interventions among this population. Following each theme, a pain and gain point is described to summarize the personal challenges faced by users and opportunities to overcome these and meet their needs through app-based interventions. The findings are also presented in Table 1. Pseudonyms are used to maintain participants’ anonymity.

**Table 1:** Summary of the pain and gain points, themes, and practical recommendations.

Consideration	Themes	Pain points	Gain points	Recommendations for future apps
Marketing	Recognizing symptoms and attitudes toward accessing help	May not feel as if they are *sick enough* to warrant attention or treatment	Focus on early intervention to educate and encourage users that they may benefit from an app	Increase awareness about eating disorder symptoms and benefits of early intervention Self-assessments can provide feedback and indicate potential benefits of early intervention Careful marketing of an app to encourage, and not discourage, potential users
Approach	Effective content that reflects key processes in recovery	Fear of losing the control that symptoms provide	Identify personal values and goals to support motivation and recovery	Incorporate reflective questioning Encourage development of more holistic self-worth Identify values and future goals
Inclusions	Social connection to facilitate hope and support	Feelings of isolation and loneliness are common	Create a safe environment, which facilitates a sense of connection and belonging	Use opportunities to help people feel a sense of connection and belonging Provide data on concurrent users and progress that can help to convey a sense of being in it together Incorporate stories and journeys from others that can give people hope
Engagement and retention	Usability and language are key	Fear of failure coupled with self-critical evaluations and low worth	Provide praise and encourage self-compassion to promote continued engagement and recovery	Use compassionate language and framing Provide a careful balance between establishing credibility and presenting content in a friendly, informal manner Make the app visually appealing and ensure that the app is easy to navigate Facilitate flexible access

#### Theme 1: Recognizing Symptoms and Attitudes Toward Accessing Help

Findings revealed that none of the participants who had been diagnosed with an eating disorder (0/7, 0%) identified with their diagnosis at the time they were experiencing symptoms. Participants did not think they were “sick enough” to warrant or deserve treatment and, on reflection, had incomplete knowledge of eating disorders and their associated symptoms. For example, media portrayals of eating disorders seemed to form the basis of their understanding of such conditions, where symptoms were perceived as more severe than their own. Furthermore, some participants appeared to normalize these behaviors, often downplaying their symptoms and severity:

I think I was 16 or 15 when I started engaging in behaviors and stuff, and I didn’t really think anything of it either. I hadn’t really read much about it or knew much about those things unless it was really severe, and I was like, whoa, that’s definitely not me. Like you see on media and stuff, where it’s all skeletons and stuff. And then I was just engaging in behaviors for a few years, but not all the time to extremes or anything.

Sophia

So then I kind of battled with it, being like, no, I can’t have an eating disorder, I’m just healthy. Maybe I’ve got orthorexia, that unhealthy obsession with healthy eating.

Sarah; after being diagnosed with anorexia nervosa

Other participants also commented that they did not want to take steps to address their eating problems. This stemmed either from fear of making changes and the daunting nature of beginning a recovery journey or from concerns about losing the connection to self and identity that their eating disorder had provided—making them feel special, important, and powerful, often for the first time in their lives:

Well, I don’t think it was that I didn’t want to admit that I had a problem, it was that I didn’t want to fix it. The anxiety around eating and gaining weight and doing all that sort of stuff you have to do and not being able to exercise and eating around people and things like that was too scary. So, it’s easier to just stay in the disorder than to do all of that.

Sophia

And feeling special, I could do something different and lots of people comment on the weight loss, self-control and that reinforces it. I knew that I wasn’t normal, but it felt powerful or special for having such good self-control or able to lose weight. All of this type of stuff. The benefits of not being normal were so regularly reinforced that I didn’t care.

Lisa

This lack of desire to address disordered thoughts and behaviors was a barrier to seeking help or support, and, concerningly, a couple of participants (2/7, 29%) admitted that they did not think they would have downloaded or used an eating disorder app around the time of their diagnosis. Instead, participants suggested that this type of app should be targeted during the early stages of the life course of body image or eating concerns, before the existing symptoms worsened:

At the start, if I’d been told earlier, I would’ve downloaded an app like that to try and help. But in the space between when I was already like that, between getting bad and getting treatment, I wouldn’t have, because I wouldn’t have accepted within myself that there was something wrong. But in treatment, if I had a psychologist or my dietician told me to download an app like that, I would have.

Beatrice

I think the app would be so beneficial really early on. And not when they’re engaging in disordered eating, or really that early start. And identifying what disordered eating looks like...I definitely think the psycho ed side of things would have been so helpful, to realize when it was going down a very dangerous track. I think that would be particularly beneficial.

Lisa

#### Pain and Gain 1: From Not Wanting Help to Acknowledgment of a Problem

It appears that this denial of a problem or ambivalence to change may act as a significant barrier to app-based eating disorder interventions (pain 1). This suggests the importance of early intervention and positioning of the app for young people early in the emergence of disordered eating attitudes and behaviors, before symptoms become very severe or the eating disorder becomes part of the individuals’ identity. The gain point that emerges from this theme is that young adults may need to be enlightened and educated about the extent of their symptoms to demonstrate that they may be in need of early intervention. For example, questionnaires may be used in marketing, screening, and tailoring of app content to highlight and reinforce the relevance and potential of such a resource to them (gain 1).

#### Theme 2: Effective Content That Reflects Key Processes in Recovery

Participants discussed a range of approaches that were helpful in their recovery journey and recommended that these could be incorporated into an early intervention app. Of the 7 participants, 6 (86%) reported that using reflective questioning to identify unhelpful thoughts or behaviors was critical for their recovery. This technique provided participants with deeper understanding of the connections between their thoughts and behaviors and allowed them to identify that their eating behavior was disordered. This practice also helped participants to see how these attitudes and behaviors may have been affecting their lives and how they may start to address it:

Mind mapping...it definitely helped, and that was a lot of what my therapy was based on and trying to find the exits from those loops, the continuous loops...Because I didn’t know that was the process going on in my head, until we actually wrote it out.

Beatrice

Understanding your thought processes about how you get to that point about feeling bad about your body...having information about how a certain image or situation can kind of...lead you to having issues with your body and issues with eating.

Alex; when asked what they would like to see in an app

Through reflective questioning in recovery, participants realized that their eating disorder stemmed from issues beyond just food or exercise. Eating and exercising behaviors were often being used to exert some control or manage negative emotions. For example, a participant described how bullying had led her to start to look for control in other aspects of her life:

Basically it began with getting some bullying in primary school and high school. High school bullying is a lot different because girls can be vicious. But I think a friendship breakdown at the start of year 10 sort of all contributed to sort of me wanting some sort of control in my life.

Rachel

In addition to identifying problematic thoughts and behaviors, participants also emphasized the need to replace these with more positive and helpful thoughts and behaviors. Providing ideas for how to think differently about the self and body was suggested as particularly valuable. It was clear that many participants (5/7, 71%) judged themselves based solely on their appearance, typically resulting in negative self-evaluations. In contrast, during and after recovery, participants had started to take a more holistic view of themselves and their bodies, which allowed them to feel more comfortable. Having a broader understanding and experience of their bodies led them to a range of helpful perspectives including aspects of positive body image, body neutrality, and enhancing self-worth:

I know that it can help people who are struggling like I was, with seeing people being comfortable in their bodies and living healthily at any weight...I thought health had to be within the BMI and what you ate and how you exercised. And seeing a lot more information, and people advocating the health at every size, that has also brought a lot of comfort in me, especially now that I’m also studying health science.

Beatrice; talking about body diversity

One thing that actually really helped me I think, [was] being actually neutral about your body and not being so black and white about it. I basically...And I always endeavor every day to just sort of see that this is what my body is and it’s fine...Like being able to sort of give resources to help people realize that bodies are not just what they look like. It’s a vessel for yourself...Your body is a lot more than what the mainstream media promotes.

Rachel

These quotes emphasize the value of focusing on alternative views of the self and one’s appearance that does not focus solely on esthetics. By doing so, participants were able to identify and value aspects of themselves that were distinct from their appearance, and this provided them with opportunities to enjoy or be proud of their body, a feeling that was new to them. Other elements that participants found to be helpful in their journey of recovery were identifying other critical aspects of their identity beyond the eating disorder, incorporating a values-based approach, and focusing on the future and potential goals:

And once I started getting back to uni and having a job, I had other things in my life that weren’t about the eating disorder and that is what started to really shift that...Once I made that decision and filled my life with things that were not related to my eating disorder, it just got so much easier.

Lisa

And just confirmation, things to repeat to myself like, is what I’m doing healthy? Is what I’m doing going to bring me a future, a good life? To make me second guess what I’m choosing to do in those moments. That has definitely helped me and trying to look towards the future.

Beatrice

#### Pain and Gain 2: Taking Control of Personal Values and Goals

In this study, young adults with body image and eating concerns frequently used food or exercise as a means to exert control, regulate emotions, or manage stress and anxiety, often when they felt that a situation or aspect of their life was out of their control. The challenge for early interventionists and app developers is that, if these behaviors become regular coping mechanisms, users may fear losing the control that they desire, which may result in a subjective evaluation of change as *risky* and reluctance to engage in intervention content through an app (pain 2). A way to address this is to leverage personal motivations by identifying values and goals that are specific to the user. By concentrating on future potential achievements and outcomes and targeting realistic goals, users may be less frightened and more motivated to initiate their recovery journey, as they know the possible benefits that are personally meaningful to them (gain 2).

#### Theme 3: Social Connection to Facilitate Hope and Support

Participants discussed a desire for an app to provide a feeling of connection. This was especially pertinent as all participants (7/7, 100%) had experienced some level of loneliness and isolation in the experience of their eating disorder. A number of participants (4/7, 57%) suggested that an app can allow the user to communicate with others, which can facilitate feelings of social support. Although some participants suggested formats within an app including chats, forums, or meetups, most participant (3/4, 75%) also acknowledged that this may be unhelpful or even dangerous if used inappropriately, and thus strict moderation will be needed:

And just having access to support systems. And also, I don’t know, like a chat room or something, where it’s just a one-on-one. And if you just feel like you really need to talk to someone there and then, and you’re by yourself, just being like, I’ll just hop online and talk to someone.

Sarah

I think being able to talk to people who are going through the same thing, or who have, or whatever, is really important, because sometimes you just have questions that no one can answer even if they are a doctor or a therapist, because they’ve never been through it. So I think having people who can help you in that way is good, but also, you have to be careful as well that they don’t say unhelpful things. Which can make it hard, because you have to have someone to moderate it, but then I feel like that might make it harder for people to say something because they know it’s being moderated. So, it’s, I guess, a fine line between what to do.

Sophia

Although existing support, such as professionals or family members, may provide some element of care, it was presumed that they will not understand the issues experienced by participants. Consequently, their opinions seemed less relevant or helpful to participants. In contrast, participants wanted to talk to and hear from others who had first-hand experience. There was some indication that individuals who had been through the same experience will have an overall different and deeper level of understanding and empathy. Participants felt that they will just “get it.” A number of positives of this lived experience perspective were identified by participants, including sharing useful strategies of how individuals dealt with certain situations and providing hope and encouragement for recovery and the future:

I think I just needed support. And I just needed someone, or people...whether it was a health professional or my friends, just to like, there is going to be an end, and at the end it’s going to be okay...So I think definitely hearing from past experiences on how people who have recovered dealt with that, would be really helpful.

Sarah; when asked what they needed when they were in their most vulnerable state

It makes you feel less alone and that you get all of these health professionals saying that it’s possible and it’s going to be hard, but you don’t actually have anyone sitting there saying I’ve done it, and it’s better. In my last admission where the nurses really got stuck into me, one of them actually had lived experience and she was also around my age and she was also in hospital for a little bit. And she was one of the people that definitely helped me get to where I was, where I’m seeing someone who has that future that everyone’s making you picture and work for.

Beatrice

And it’s proof when you’ve got so much proof on social media, and I guess in your own head that what you’re doing is fine. When you’ve got someone saying, no, I was in that mindset, it wasn’t fine, this is better. That is one of the most important things to me.

Beatrice

It appears that this shared lived experience may be an especially powerful aspect of recovery. The idea of hearing from others who had experienced the same concerns also facilitated some level of hope for participants. Participants felt that being able to hear from these people will provide some sense of trust in recovery—that others have done it and that there is light at the end of the tunnel. Having this alternative route and point of view can keep users motivated, knowing that, although it is hard, it is both possible and worthwhile. Seeing a role model may encourage users to achieve this for themselves.

#### Pain and Gain 3: Moving From Loneliness to a Sense of Belonging

Although intense feelings of loneliness meant that participants desired the ability to communicate with others, regarding an app, this would be challenging to implement in such a way that it would not cause harm (pain 3). The opportunity from this is that, if app developers can provide helpful content and facilitate a feeling of connection and belonging through an app, this may help users to turn off their inner voice and move away from disordered patterns of thoughts and behaviors. Ultimately, the content, approach, and language used in an app can promote a feeling of belonging and an element of safe social connection, while providing evidence-based and accurate information. This may help users to begin to feel more motivated to engage in the content and develop a sense of hope that they can and will recover (gain 3).

#### Theme 4: Usability and Language Are Key

The usability of the app appeared to be very important for participants, including aspects such as content readability and app accessibility. Participants expressed a desire for an app that is intuitive and easy to use. Relatedly, the look and feel of the app are critical factors that likely affect uptake and retention over time. A participant described their experience of using a similar mental health app:

Well, I know there’s one called [eating disorder app name removed for anonymity], which I used with the dietician for a little while. I think its functionality was clunky...it was hard to use and didn’t really look that great, so...functionality and look and stuff is probably something that would make a difference to whether I would download it or not if it popped up.

Sophia

But I also think just in terms of access, making things really easy to get through on the app. Rather than a lot of words, because that’s really off putting and I definitely wouldn’t have like that as a 16 year old. Something really easy to use and not too wordy, or theory based, or jargon based.

Lisa

Participants also had the expectation that an eating disorder app should be developed by experts and professionals to ensure its credibility, reliability, and effectiveness and that this would make them more inclined to spend time on an app. This could be achieved by the use of research, connections to universities, and having health professionals directly refer people to the app. The language used within apps also seems to play an important role here, specifically ensuring that users both trust and understand the information being presented and do not feel overwhelmed by it. Achieving this professional yet informal balance will likely encourage continued use and engagement:

I would be using it as a support and as a friend, so I would want it...the language and stuff to be used, very informal, chatty...Just because then it’s not as scary. It’s all based on studies and everything, [but has] some sort of informality to it.

Sarah

I think it being suggested to me by health professionals would have been something that I would have been like, okay yeah, I’ll give it a whirl. I think I’d have more trust, and I’d feel safer using it.

Sarah

I think if it was promoted in a very compassionate way like, eating is allowed to be enjoyable...You’re allowed to feel this way, your body isn’t just what you look like. But having it promoted with language in that sort I feel like if that was the case then I might’ve looked into it. I just think it has to have a lot of sort of compassion focus.

Rachel

In comparison with face-to-face support, a key advantage that an app provides is the flexibility and freedom for the user to engage at any time. Participants described how an app should have the ability to be used fluidly to suit the needs of the user:

I think it has the potential to be really useful and be a success. But it’s down to the individual, that’s the only thing. I think having an open module with no time limits or anything, that would be preferred by me because there were some days where I didn’t have the mental strength to focus on anything.

Sarah

So it’s not so much of a structured application or process within the app. I think that would be really appealing for people who were worried about having to commit to something and knowing that they can always, not opt out, but they can always just come back into it.

Alex

Participants also indicated that it was important for apps to be personalized or tailored to users’ needs. Participants perceived that an app of this nature will require hard work and effort. They described fear of commitment to a long program and concerns regarding the mental capability needed to work through the app content. The emphasis on flexibility suggests that an app will be used more sporadically, rather than, for example, scheduling a certain time to engage each week. The expressed preference for fluidity within an app may also indicate that some users may have difficulty in maintaining motivations or that they only feel moderately committed to working through an app. Aspects that may help to maintain motivation and retention are personalization and relevance for the user within the app and providing some features throughout the app that allow for participant input, including the ability to write ideas, comments, or responses directly into the app and check-in questions for the user:

I feel like when you can input, like the little faces to check Very Good. I like that. It’s also, you can record audio messages, put in photos. I think that’s great. And then down the bottom it says like, how much sleep did you get, how much exercise did you do, and how well did you eat? You can just input. I think that’s a really good tool.

Claire

I struggle, like [to] be consistent. That’s my personal thing. I might do it for a few days and then forget to do it. So like, whether I remind the system or something that would be useful, but that kind of thing, or say you could find a friend to be like, “Oh, have you checked in on the app today?” That kind of accountability thing.

Claire

It appears that users may find continued use of an app to be challenging, including maintaining motivation over a prolonged period. Given these concerns, participants welcomed and seemed open to the idea of reminders or check-in notifications used within the app to prompt use. This may also make users more accountable for their engagement.

#### Pain and Gain 4: Addressing Fear of Failure Through Encouragement and Compassion

Participants perceived that an app will require significant effort, meaning that some will prefer to use it more sporadically (pain 4). From the empathy mapping process, it appears that this sample typically demonstrated some perfectionistic and competitive qualities and tended to be high achievers, meaning that they often strived to be the best. Therefore, it is likely that they fear feelings of failure when using the app and may be extremely self-critical. In contrast, participants craved and thrived on acknowledgment and praise for their efforts, which some felt that they did not receive during their illness. These types of prompts and rewards can be easily incorporated into apps to foster consistent use and combat lapses, thus ensuring progressive recovery. Given these perfectionistic traits, it may also be important to model a self-compassionate approach and remind users that recovery is neither linear nor straightforward (gain 4). If end users are reminded that setbacks are normal and encouraged to return after periods of less engagement, they may be less likely to drop out or lose interest in an app.

## Discussion

### Principal Findings

Web-based and app-based psychological interventions offer some advantages to delivering early interventions and treatment by overcoming barriers related to cost, time, and location and concerns regarding the lack of anonymity and perceived stigma of treatment seeking. However, difficulties such as low uptake and retention remain. Using a novel design thinking approach, this study aimed to provide insights into the lived experience perspectives of potential end users regarding app-based interventions for eating disorders, including identifying key challenges and opportunities. Several issues arose from these insights. First, people with eating disorder will need to recognize that they have a problem that is sufficiently serious to warrant treatment and want to seek help for them to engage with an app. Second, specific content for the app, including processes that enable users to record their experiences, was suggested to facilitate recovery. Third, there was a strong desire for an app to facilitate social connection and sense of belonging. Finally, app usability was identified as important, including the use of compassionate and supportive language and setting a tone that is both credible and friendly.

### Comparison With Previous Studies

The first issue that became clear in reaching end users was that many may not know that they have a problem or want help. Individuals with lived experience reported that they would likely not have engaged with this type of app when their eating disorder was most severe or established. As found in previous studies, participants did not believe that their problem was sufficiently serious to warrant treatment [37,38] or they feared losing control [9]. A key challenge for the implementation of an early intervention app is identifying and engaging with those in need, encouraging them to recognize that need, and informing them that they can benefit from using an eating disorder app. To address this, when marketing and disseminating this resource, researchers should aim to increase awareness and understanding and challenge inaccurate perceptions of eating disorders through psychoeducation, including reference to the broad range of conditions and symptoms. In support of this, a recent Australian study also found that 87% of participants reported a preference for psychoeducation and screening scales to assess symptoms within app-based interventions [16]. Another critical consideration is the framing and *sales pitch* of an app. Researchers developing apps need to think carefully about who the app is intended for and how to engage potential users. For example, is an app intended to be used regularly as a form of treatment to replace or complement therapy, or does it aim to provide in-the-moment support that can be used sporadically? [39] Understanding this and marketing an app accordingly will provide clear expectations to the user, likely resulting in higher retention over time.

Participants identified some particularly helpful activities or perspectives that they experienced, which facilitated their recovery, and recommended that these be incorporated into app content, including verbalizing, writing, and visualizing thoughts, attitudes, and behaviors. These are common in practices such as cognitive behavioral therapy and in other mental health apps, which may theoretically translate well into an app context. A recent systematic review of evidence-based treatment elements in eating disorder apps found that cognitive restructuring was included in only 21% of apps, despite 56% of active monthly users using this function when it was included, making it one of the most frequently used features [40]. In addition, consistent with studies on positive body image, participants in this study also reported content that encouraged more holistic views of beauty and worth as being helpful [41]. Furthermore, the fear of losing control that disordered eating behaviors can cause may be overcome by focusing on the future and identifying personal goals and more meaningful achievements than those associated with weight and appearance. Other studies also suggest that combining goal setting with daily reminders is associated with great app use [42]. This appears to be a helpful strategy to disrupt mechanisms that may maintain eating disorder symptoms, thus making users more open and willing to change.

Given the loneliness and isolation reported by all participants (7/7, 100%) at some stage of their eating disorder, it is perhaps unsurprising that there was a strong desire for an app to facilitate social connection and hope. This may also be useful in helping users to overcome their critical inner voice and allow them to hear alternative views or perspectives. Although a key obstacle of app-based interventions is the lack of human contact or reciprocity [43], participants in this study provided some suggestions to combat this, including communication with other app users through a chat or forum. However, given that this may be accompanied by a range of challenges and potential dangers for users, researchers can instead focus on using innovative and creative thinking to identify alternative ways to reduce feelings of loneliness and enhance a sense of belonging through an app, without direct communication. For example, this can be achieved with inclusive and welcoming language and may include providing notifications or messages to users to indicate that other users are also using the app currently (eg, “[number] other users have been online this week/are now online”). It is possible that an e-therapeutic alliance can also be achieved in digital health interventions through user engagement and careful design choices [44]. In addition, using recovery stories from individuals with lived experience may facilitate feelings of hope for users. Future technology may also find a way to facilitate social connection, including chatbots or moderation for a chat room–type function [15], as has been done in other areas of mental health research [45]. Although these features can be incorporated into an app, they can also be offered as adjunct supports for people with eating disorder.

The final theme highlights the key role of app usability in participant uptake and engagement. Given that poor usability has been found to predict nonadherence to web-based interventions [46], this finding is an important reminder for researchers to focus considerable efforts on app accessibility, including the need for continued and rigorous testing with end users and design approaches to identify and address issues. The language used within an app itself is also important. End users may fear failure and be especially self-critical. Therefore, ensuring compassionate language that emphasizes the nonlinear journey of recovery and praise when completing activities and progressing through an app may be helpful. Studies suggest that self-compassion may be an effective intervention target, more so than alternatives such as mindfulness [47]. Relatedly, the language and tone of an app need to strike a careful balance between appearing credible (ie, created and recommended by professionals) and being personable and informal (ie, similar to talking to a friend and using language that is understandable and relatable). In addition, it appears that users want an app that can be used flexibly to fit around themselves and their lives. These findings highlight how the look and feel, functionality, features, and usability of an app are extremely important, thus indicating that the time invested in making an app appealing and intuitive during its development is critical [21].

App features that emphasize personalization were mentioned favorably by participants, including allowing users to enter their own input (eg, writings and audio messages) and track their mood. Consistent with this, qualitative studies exploring the usability of an existing eating disorder app found that participants value being able to monitor their progress and felt that it improved their self-efficacy and motivation to use the app [43]. Although some personalized elements may be easy to implement (eg, notifications, prompts, or branching of content and feedback based on early user inputs), others will be more complex and require expertise to be incorporated into an app (eg, use of chatbots to enable an ongoing, personalized dialogue with users). It is also important to emphasize that users and designers may have different interpretations of *personalization*. For users, this may be about feeling that the app is responsive to their inputs and not simply providing generic scripts and videos that fail to pick up on information that may make subsequent content unnecessary. In contrast, programmers may think of personalization in terms of more complex design elements, such as chatbots. Given the rapid evolution of technology, it is important for researchers to be up to date with newly emerging programming that may produce advancements for app personalization. However, it is also important to note that this high-level personalization may be beyond the scope and capacity of most academic-led projects and may not always be desired or necessary for establishing an engaging and efficacious product. This reinforces the importance of end user testing, co-design elements, and ensuring that researchers are clear in their interpretation of user feedback.

### Limitations

This study used a novel, design thinking approach to provide better understanding of the needs and preferences of potential end users of app-based interventions for eating disorders. These findings can be used to inform the development, framing, and dissemination of eating disorder apps. However, the study has a number of limitations. First, although this study identified several insights into strategies to enhance uptake and retention, the retrospective nature of these data means that suggestions are informed by hindsight and may be different from what participants may have said if interviewed in the early stages of their illness or during their treatment. Second, the present sample was limited to women and may have been moderately biased in that they self-selected to participate, indicating an openness to discussing eating disorders and engaging in research. However, this study was exploratory in nature and the sample was not intended to be representative. Nonetheless, the authors encourage the use of these methodologies among large samples. Finally, even in this small sample, there were some slight inconsistencies in participants’ preferences, which highlighted individual differences. For example, some participants placed more emphasis on social support or journal writing within an app than others. Therefore, further studies will be helpful to unpick some of these nuances to better understand individual preferences among users.

### Implications

There are a number of important practical and clinical implications of this study. Researchers developing theoretically informed and evidence-based apps must consider the needs of the target group when developing resources. This study may be used to inform app developers or researchers who may not be familiar with the lived experience of an eating disorder and those trying to put themselves back into an early intervention mindset if they have not experienced this recently. In addition, the pain and gain points can be used to directly inform the design and development of such apps. Relatedly, it is critical that researchers explore and identify key drivers of uptake, which may inform users’ willingness to change. Consideration and implementation of such practices will likely enhance app uptake, engagement, and retention, with the potential to support individuals who may not otherwise seek or access eating disorder information elsewhere. Importantly, cost evaluations that examine the impact of various design and implementation features should be performed to ensure that they are appropriate and effective.

### Conclusions

This study extended previous research by using a novel design thinking approach to understand and empathize with individuals with lived experience of eating disorders to identify their needs and preferences for app-based early interventions. Although a number of challenges are experienced by this population, including feelings of loneliness and ambivalent attitudes toward change, this study has identified some opportunities that may be considered in the design and development of an eating disorder app. These include helping users to develop insights into eating disorders, identifying personal values and goals to encourage behavior change, providing elements of social connection to facilitate hope, and ensuring that the language and framing of an app is appropriate. Researchers and app developers should consider these aspects to develop future early intervention apps for women in ways that enhance app uptake and retention and improve eating disorder symptoms.

## Appendix

Multimedia Appendix 1 Semi-structured interview schedule.

Multimedia Appendix 2 Overview of common elements across the empathy maps.

## Abbreviations

WIRED: Web-Based Interventions to Reduce Eating Disorders
