# Supplementary material for: Informing mHealth and Web-Based Eating Disorder Interventions: Combining Lived Experience Perspectives With Design Thinking Approaches
Source: JMIR Form Res. 2022 Oct 31;6(10):e38387. doi: 10.2196/38387 (PMC9664336; doi:10.2196/38387)
Supplement: Multimedia Appendix 1 [file formative_v6i10e38387_app1.docx]

# Multimedia Appendix 1

# Semi-Structured Interview Schedule

1. I just wanted to get to know a little bit more about you as a person and as much as you're comfortable telling me today, I'd like to hear your personal story of how disordered eating has affected you (including follow-up questions and prompts to extend the discussion)
2. How do you think social media influences your body image?
3. What do you think could be done to change the way that social media influences young people’s wellbeing? And is there anything that you think wouldn’t be helpful?
4. If you were going to develop materials to improve body image and eating issues, what would they look like, what would they focus on, and what would they be called?
5. How do you think that you could use an app to support your body image concerns or eating behaviour?
6. What would attract you to use or access such an app- how would it be described?
7. What are some of the features that you might like to see in an app to support eating behaviour and body image?
8. Can you describe any sort of self-directed (e.g., books), online, or/and professional support that you have accessed in relation to body image and eating behaviours?
   - What did you liked/ what worked about that support?
   - What didn’t you like/ what didn’t work?
