# Supplementary material for: Informing mHealth and Web-Based Eating Disorder Interventions: Combining Lived Experience Perspectives With Design Thinking Approaches
Source: JMIR Form Res. 2022 Oct 31;6(10):e38387. doi: 10.2196/38387 (PMC9664336; doi:10.2196/38387)
Supplement: Multimedia Appendix 2 [file formative_v6i10e38387_app2.docx]

# Multimedia Appendix 2

# Overview of the Common Elements Across the Empathy Maps

### Who are we empathising with?

All participants were diagnosed or identified their own body image or eating issues as a teenager or young person, with symptoms typically starting as body image concerns before spiralling to more serious issues with food or exercise. The majority (*n* = 5) described some major life event or change which coincided with the development of their eating disorder, including bullying at school or starting university. Four of the participants reported having perfectionistic tendencies and were often high achieving such as in high school and sports performance (e.g., netball or running). Three grew up in small, regional towns which introduced challenges in seeking specialised support.

### What did they see?

When asked where they were seeking body- or eating-related content and information, the majority reported using social media (*n* =5; e.g., Tumblr, Instagram, Pinterest). This space seemed to work as a bubble or echo chamber, reinforcing their disordered thoughts and behaviours as normal or healthy as opposed to problematic. Interestingly, three of the participants went on to later study nutrition at university. These participants described the conflicting information that they started to receive, and noted that the evidence-based and health-focused perspective they were starting to see at university was starkly different to the information online through social media, which often promoted and glorified strict diets and exercise regimes. While learning about nutrition at university was a new space to navigate, it helped participants think differently about food, considering the nourishing and functional aspect rather than using food as a coping mechanism or to modify the body’s appearance.

With regards to help seeking, five participants described how they saw people around them trying to help them to access support, even though they did not want, or think they needed to seek help themselves. This appeared to have a toll on their families, particularly their parents, with two participants describing how they saw some people lose energy and momentum for their recovery (e.g., “my entire treatment team had given up, my family had given up, no one thought that I was going to get better”).

### What did they say and do?

At diagnosis, and even when symptoms were particularly severe, all of the participants expressed denial that they were experiencing an eating disorder. They did not identify with having an eating disorder and did not want help, downplaying their symptoms and believing that there were likely others who were ‘sicker’ and had it worse than them. All participants frequently used food or exercise as a means of coping with negative emotions or experiences, to gain a sense of comfort and/or control within their situation, or to punish oneself. Two participants talked about the secrecy of eating disorders and their attempts to hide their symptoms from others, particularly during treatment. Most participants (*n* = 5) frequently engaged in comparisons and competitive thoughts (e.g., waist measurements, number and quality of compliments received), with both self and others, and negative self-talk was common among six participants.

### What did they hear?

Six of the seven participants spoke about hearing their own voice inside their head. On one hand, it was criticising them, telling them that they were not good enough as a person and that they need to be better (e.g., ”Oh, why did you do that? That was such a big mistake”). On the other hand, for some, it simultaneously encouraged the eating disorder and related behaviours (e.g., [referring to losing weight] “The voice in me said that's good, people are noticing”). Most participants (*n =* 4) also had those around them, primarily friends or peers, reinforce their eating disorder symptoms through praise (e.g., “you look great”, “you’re such a fast runner”). Two participants admitted that they didn’t often experience praise or positive feedback from others, so for them, when praise was given for eating disorder related symptoms or behaviours, it strongly encouraged and increased those disordered eating behaviours. Despite this reinforcement, it was concern expressed from those around the participants about their health and well-being that eventually led five participants to seek support and treatment. Participants reported getting messages from loved ones that something needed to be done, with the responsibility for action either placed on the participants directly (e.g., “*you* need to put on weight”) or they were told that others were taking control of the situation on their behalf (e.g., “*we* are admitting you to hospital”).

### What did they think and feel?

As a result of continued comparisons and self-criticism, participants reported having very negative self-evaluations including low self-esteem and self-confidence and negative mood. While some (*n* = 3) reported good relationships with close others (e.g., mother, friends), some (*n* = 3) experienced difficulties building social connections, often feeling like they didn’t fit in. As well as negative self-evaluations, they also thought that others judged them or didn’t care about them. This resulted in all of the seven participants feeling lonely and isolated through their experience. Participants also felt confused because of conflicting messages between their internal thoughts (e.g., “I’m not sick enough”, “I need to be thinner”), what they were seeing around them (e.g., weight loss tips on social media), and what they were hearing from others (e.g., “you are seriously ill” vs “you are not ill enough”; “you look unwell” vs “you look great”).
